# Sequencing of One Unique Recombinant CRF85_BC/CRF01_AE Genome and Two Partial Genomes from Ningxia, China

**DOI:** 10.3390/v17050655

**Published:** 2025-04-30

**Authors:** Yufeng Li, Jianxin Pei, Xiaohong Zhu, Yichang Liu, Xiaofa Ma, Dongzhi Yang, Zhonglan Wu

**Affiliations:** 1 School of Public Health, Ningxia Medical University, Yinchuan 750004, China; liyf0909@163.com (Y.L.); zxh_00921@163.com (X.Z.); yichang_cc@163.com (Y.L.); xiaofa_ma@163.com (X.M.); 2 Ningxia Center for Disease Control and Prevention, Yinchuan 750011, China; peijianxin282@163.com (J.P.); nxcdcydz@126.com (D.Y.); 3 College of Life Sciences, Ningxia University, Yinchuan 750021, China

**Keywords:** HIV-1, second-generation recombinants, near full-length genome, phylogenetic tree, recombination breakpoint analysis

## Abstract

The recent emergence of new HIV-1 recombinant strains presents a new challenge to the control of HIV-1/AIDS and the development of an effective vaccine. We employed a near full-length genomic sequence analysis of a newly identified CRF85_BC recombinant strain in Ningxia, China, to determine its recombination pattern. Blood samples were collected from HIV-infected or AIDS patients in Ningxia in 2023. CRF85_BC subtype strains were detected from three samples using an in-house method, and one sample’s near full-length genome sequence was also obtained. MEGA11, jpHMM, and Simplot software were used to identify subtypes and analyze recombination patterns. Neighbor-joining phylogenetic tree analysis showed that HIV-1 pol region sequences of three samples were CRF85_BC subtypes. One near full-length genome sequence of the recombinant strain was obtained, and jpHMM preliminarily judged that the recombinant strain was inserted with two subtype B fragments and two CRF01_AE fragments based on subtype C as the backbone. Further analysis using Simplot software revealed that the recombinant strain was the second-generation recombinant strain of CRF85_BC and CRF01_AE, and the recombination mode was based on the full-length genome of CRF85_BC, and CRF01_AE gene fragments that were inserted at positions 7365–8279 and 8431–9492, respectively. The results of the fragment phylogenetic tree verified its accuracy. One CRF01_AE and CRF85_BC second-generation recombinant strain was found in HIV-1 infected people in Ningxia, indicating that new HIV-1 recombinant strains continuously emerge and circulate in this region. Genomic surveillance of these recombinants should inform targeted interventions, such as prioritized contact tracing, to mitigate the formation of transmission clusters.

## 1. Introduction

Acquired Immune Deficiency Syndrome (AIDS) is the most severe form of immune deficiency caused by Human Immunodeficiency Virus (HIV). The most prevalent HIV in humans is HIV-1, which exhibits a high degree of variability, rendering it susceptible to mutation and recombination, which leads to the emergence of series of circulating recombinant forms (CRFs) and unique recombinant forms (URFs). According to the HIV database of Los Alamos National Laboratory [1], 158 CRFs had been identified globally as of 14 October 2024. This extensive genetic heterogeneity presents significant challenges for diagnostics, antiretroviral therapy (ART), and vaccine development [2].

In China, the predominant HIV-1 subtypes include CRF01_AE (32.1%), CRF07_BC (39.1%), and CRF08_BC (9.2%) [3]. The prevalence of CRF07_BC has been increasing, while that of CRF01_AE has been decreasing over recent years [3]. In the Ningxia region, studies have focused on the genetic characteristics of HIV-1 strains, particularly the pol gene segment, which is crucial for understanding transmission dynamics and drug resistance patterns [4].

The identification of recombinant forms is essential for tracking the evolution of the virus, optimizing treatment strategies, and informing public health interventions [3]. In this study, we subtyped pol region sequences of three newly discovered HIV-1 epidemic recombinant strains in Ningxia in 2023 and determined the recombination pattern using one near full-length genome (NFLG) sequence. The aim was to provide data support for the study of HIV-1 genetic diversity and further epidemic prevention and control.

## 2. Materials and Methods

### 2.1. Sample Source

Plasma samples were collected from all newly diagnosed HIV-1/AIDS patients in the Ningxia region in 2023. In this study, all samples from HIV-1 patients were obtained from the designated collection sites, and all patients provided written informed consent for subsequent analysis.

### 2.2. RNA Extraction and Amplification

Plasma RNA was extracted using the fully automated nucleic acid extraction and purification instrument along with its matching reagent kit produced by Zhuhai Livzon Diagnostics Inc., Zhuhai, China. Based on the research by Liu YC et al. [5], nucleic acid was extracted from plasma, followed by cDNA amplification and sequencing. The HIV-1 pol region, including the full-length protease and the first 300 amino acids of the reverse transcriptase gene, was amplified using an in-house method [6,7], yielding a product of approximately 1100 bp. The amplification products were verified by 1% agarose gel electrophoresis and subsequently sent to Beijing Novogene Bioinformatics Technology Co., Ltd., Beijing, China for purification and sequencing. Three cases (labeled NX01, NX02, and NX03) were identified to be new recombinant strains and one of them, sample NX01 (HIV viral load >10,000 copies/mL and sample volume >500 µL) was selected for near full-length genome sequencing by Beijing Dehong Changyuan Biotechnology Co., Beijing, China.

### 2.3. Sequence Analysis

The obtained and sorted sequences were uploaded to the HIV databases website using the HIV BLAST online tool (https://blast.ncbi.nlm.nih.gov/Blast.cgi (accessed on 26 April 2025)). The HIV-1 subtypes were subjected to preliminary analysis, and MEGA11 software was employed to construct the neighbor-joining phylogenetic tree (bootstrap value = 100%, Tamura Nei model was utilized) for synchronous confirmation.

To analyze HIV-1 recombination, the jpHMM and Simplot3.5.1 software tools were used. The jpHMM tool, which is based on a Hidden Markov Model, identifies recombination breakpoints by comparing the test sequence to a reference isoform. However, the program has not been updated to accommodate CRF85_BC or other subsequently reported recombinants. Simplot, on the other hand, enhances the results of jpHMM by showing local sequence homology and confirming recombination sites using sliding window analysis. The use of a small window size (200 bases) is essential. In the event of a discrepancy, Simplot results were taken as the ultimate conclusion. This combined technique guarantees precise detection of recombination events. Finally, the website’s recombinant drawing tool was used to visualize the mosaic structure and gene recombination breakpoints of NFLG.

To identify recombination breakpoints and construct fragmented phylogenies, phylogenetic trees were constructed for nine HIV groups (A-D, F-H, J, K) using reference sequences sourced from the HIV database website. This approach allowed us to accurately determine the genetic characteristics of the fragments and their evolutionary relationships in the genome.

## 3. Results

### 3.1. Demographic and Genetic Subtype Analysis

**Figure 1:**
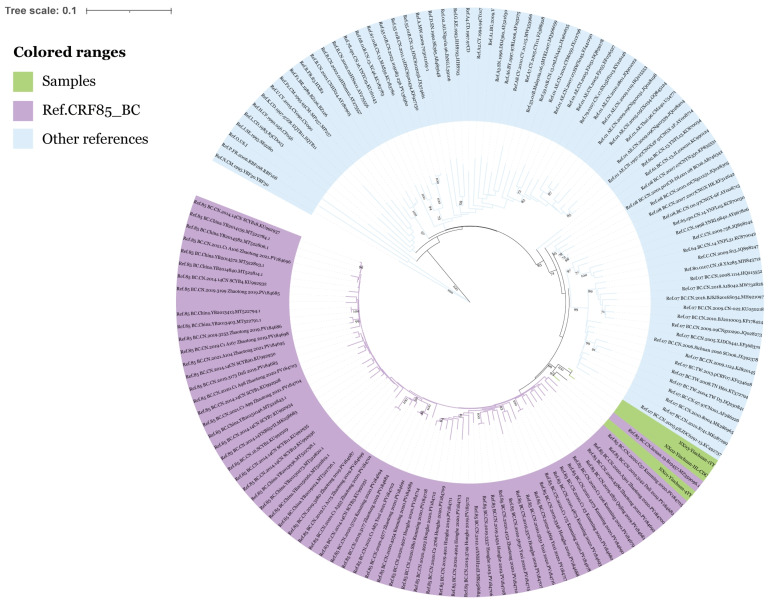
Phylogenetic tree analysis of pol region sequences from three HIV/AIDS patients in Ningxia in 2023 (green), reference sequences for CRF85_BC subtype (purple), and reference sequences for other subtypes (blue).

The demographic and genotypic characteristics of the study population were obtained from a previous study conducted by LIU YC et al. [5]. The majority of participants were male (86.64%), aged 26–49 years (58.02%), and from Yinchuan City (53.05%). In terms of marital status, 39.31% were married or had a spouse, and 34.73% had a junior high school education. The primary mode of HIV transmission was heterosexual contact (62.60%). Genotypic analysis revealed 11 subtypes, with CRF07_BC (37.79%) and CRF01_AE (35.50%) being the most prevalent. Based on the pol region sequences and the neighbor-joining evolutionary tree of the reference sequences, the reference sequences incorporated only the Asian prevalent subtypes. Three samples were identified as new recombinant strains, subtyped as HIV-1 CRF85_BC, with high homology (bootstrap value > 90%) to strains from Henan region of China, as shown in Figure 1, suggesting that there may be a trans-regional transmission pathway. All three patients were male, Yinchuan City residents, and infected through heterosexual contact. Their ages were 73, 68, and 73 years old, respectively. In addition, a more comprehensive phylogenetic tree was constructed by including more globally reported HIV-1 strains. The three samples clustered with the CRF85_BC reference strain, clearly demonstrating the evolutionary position of the studied strains within the HIV phylogenetic framework (see Supplementary Material).

### 3.2. NFLG Recombination Patterns Analysis

**Figure 2:**
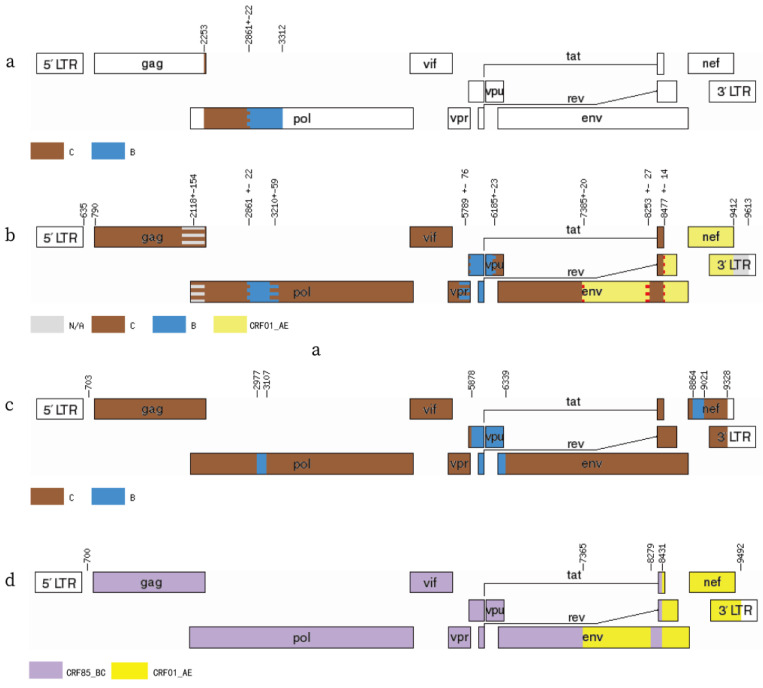
Recombinant pattern analysis of the new recombinant strain of sample NX01. (**a**) Sample NX01 pol region jpHMM recombination pattern analysis; (**b**) sample NX01 NFLG jpHMM recombination pattern analysis; (**c**) CRF85_BC NFLG reference jpHMM recombination pattern analysis; (**d**) Sample NX01 NFLG mosaic structure and gene recombination breakpoint analysis. Color Notes: C subtype (brown), B subtype (blue), CRF01_AE subtype (yellow), CRF85_BC (purple).

The jpHMM results of pol region sequences of sample NX01 indicated that NX01 was recombined by B and C subtypes (Figure 2a). Based on the gene loci of the HXB2 reference strain, the NFLG sequence of NX01 in the jpHMM demonstrated that the C subtype constituted the backbone, with two fragments likely originating from recombination with B subtype, one fragment inserted at loci 2861 ± 22~3210 ± 59 and another at 5789 ± 76~6185 ± 23, along with two fragments likely originating from recombination with the CRF01_AE subtype, one fragment inserted at loci 7385 ± 20~8253 ± 27 and another at 8477 ± 14~9412. Therefore, we propose that NX01 may be a recombinant of B, C, and CRF01_AE subtypes (Figure 2b). It is worth noting that the reference strain CRF85_BC is a recombinant form of subtypes B and C (Figure 2c).

**Figure 3:**
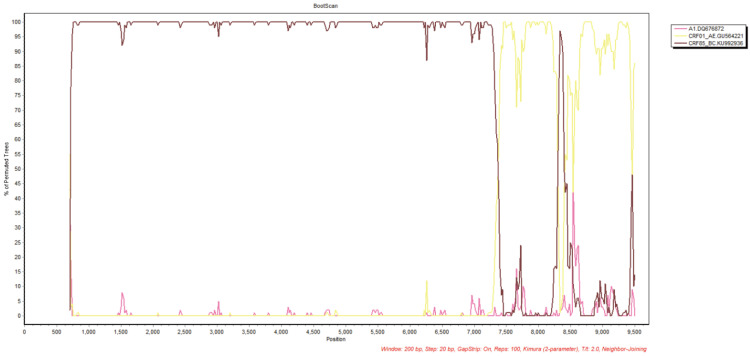
NX01 NFLG SimPlot gene similarity analysis. Color Notes: CRF85_BC subtype (brown), A1 subtype (pink), CRF01_AE subtype (yellow).

Currently, the reference sequence libraries of jpHMM online tools only encompass the A-L and CRF01_AE subtypes, and B/C recombinations are not yet included. Consequently, the Simplot3.5.1 software was employed to examine the genetic similarity between NX01 NFLG and the standard reference strains of diverse subtypes, in order to identify the locations of their recombination breakpoints. The results demonstrated that NX01 is a novel second-generation recombinant strain formed by CRF01_AE and CRF85_BC. The gene recombination pattern of this sequence was characterized by the insertion of two CRF01_AE fragments into genomic backbone of the CRF85_BC subtype. Furthermore, BootScan mapping revealed that the insertion recombination site was located at the env region of the 7365~8279 locus, with a second site at 8431~9492 (Figure 2d and Figure 3).

### 3.3. Traceability Analysis

**Figure 4:**
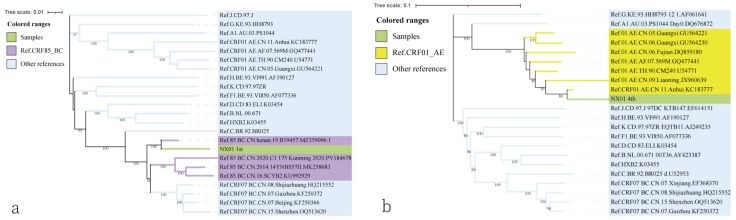
Analysis of the phylogenetic tree of sample NX01 subgenotypes. (**a**) Phylogenetic tree analysis of the first subgenotype fragment (6660 bp) of NX01; (**b**) phylogenetic tree analysis of the fourth subgenotype fragment (1260 bp) of NX01. Color Notes: Samples (green), other references (blue), CRF85_BC (purple).

The feasibility of phylogenetic tree analysis based on fragments is contingent upon the length of the gene fragments. In this study, we performed this analysis on two relatively long fragments. Information to identify the first and fourth segments by their location in the genome, in the phylogenetic tree, the first fragment (666 bp, loci 700~7365) of NX01 clustered with the reference strain CRF85_BC, showing a high level of homology (bootstrap value >90%) with virulent strains from the Henan region of China (Figure 4a). Similarly, the fourth fragment (1260 bp, loci 8431~9492) of NX01 clustered with the reference strain CRF01_AE (bootstrap value = 89%) (Figure 4b), suggesting that a possible transmission pathway across regions.

## 4. Discussion

As the global AIDS pandemic continues, HIV genes are evolving and new recombinant strains are emerging. According to the latest data from the Joint United Nations Programme on AIDS (UNAIDS) [8], 1.3 million people were newly infected with HIV in 2023, with women and girls accounting for 44% of all new infections. HIV prevalence among the five key populations, men who have sex with men, people who inject drugs, sex workers, transgender people, and people in prisons and other closed settings—remains significantly higher than that in the general population. The HIV databases website [1] shows that a large number of epidemic recombinants have been identified due to recombination between HIV-1 subtypes, with the latest novel epidemic recombinant being CRF158_0107. China’s national molecular epidemiological survey revealed a significant increase in the number of CRFs and URFs with the change in transmission routes [9]. While the prevalence of subtypes CRF01_AE and B is decreasing, the proportions of CRF07_BC, CRF08_BC, CRF55_01B, and other CRFs and URFs are increasing [10]. In Ningxia, CRF01_AE and CRF07_BC have been the main prevalent genotypes in recent years [11], with CRF55_01 and CRF65_cpx being detected as sexually transmitted in men, with the rate of heterosexual transmission being higher than that of same-sex transmission [12]. In this study, three new cases of the CRF85_BC strain of HIV-1 were detected in Ningxia in 2023, all of which were in male patients and transmitted through heterosexual means, indicating the emergence of high-risk groups for novel recombinant strains.

To accurately determine the recombination pattern of a new strain, it is necessary to analyze its near full-length genome sequence. The first-generation of HIV sequencing is limited in scope as it can only detect known genomic regions or specific gene sequences. In contrast, second-generation sequencing can analyze the entire genome or exome, making it suitable for genetic variant screening and detecting inferior strains in complex quasispecies with ultra-high sensitivity. The subtyping of HIV-1 is typically based on the partial pol sequence, which may affect the accuracy of determining recombinant subtypes. The total length of the HIV-1 genome is approximately 9.7 kb, and in the initial phase of this study, the sequenced region of the pol gene was only 1.1 kb in length. Therefore, the recombination results obtained from the pol gene sequence may lack accuracy and completeness. This study found that the recombinant strain had fragments inserted in the env, rev, and nef genes. The env gene, which encodes the membrane protein (especially gp120 and gp41), is a crucial site for viral binding to host cell receptors. As a result, the env gene is highly susceptible to recombination and contributes to the significant variability of HIV-1.

The CRF85_BC strain identified in this study is a second-generation recombinant derived from CRF85_BC and CRF01_AE, underscoring the dynamic genetic diversity of HIV-1 in China. CRF01_AE was introduced into China in the 1990s through heterosexual contact from Thailand and initially became prevalent among heterosexual individuals and drug users in Yunnan and Guangxi [13,14]. As the primary mode of HIV transmission shifted from blood and injecting drug use to sexual contact, CRF01_AE rapidly disseminated to broader populations, creating favorable conditions for the emergence of second-generation recombinants [15]. The insertion of CRF01_AE fragments into the CRF85_BC genome suggests that its recombination process may have involved superinfection, wherein a host, already infected with one HIV strain, is re-exposed to another strain, leading to further viral genome mutations and recombination [16]. Phylogenetic tree analysis (Supplementary Figure S1) confirms that CRF85_BC clusters with known CRF85_BC reference strains, solidifying its evolutionary position within the HIV-1 phylogenetic framework. It is hypothesized that the Ningxia CRF85_BC/CRF01_AE recombinant strain may have formed in the mid-2010s (ca. 2013–2018), based on the prevalence pattern of HIV-1 recombinant strains in China and the timing of CRF85_BC reports (post-2016 [17]). This recombination is indicative of the mixing of CRF01_AE (introduced in the 2000s) with the later-emerging CRF85_BC in the northern transmission network, a process that may have been facilitated by cross-provincial population movement or localized high-risk behaviors (e.g., men who have sex with men, MSM). This observation is consistent with the trend of the evolution of the second-generation recombinant strains (post-2010) in China.

Our research group has been continuously monitoring the HIV-1 sub-types in Ningxia, providing annual updates on genetic diversity and emerging recombinant strains. As a result of this ongoing effort, we were able to identify the presence of CRF85_BC in Ningxia in 2023, marking its first detection in this region. The emergence of new recombinant strains, such as CRF85_BC, presents significant challenges for public health measures, as they can affect the accuracy of diagnostic tests and the effectiveness of antiretroviral therapies. These findings highlight the importance of sustained surveillance and strategic adjustments to address the evolving HIV-1 epidemic [10].

HIV molecular traceability technology is a hot spot in current molecular epidemiological research, which can trace the source of infections and speculate on transmission routes. The results of the pol-region phylogenetic tree in this study suggest that the emergence of the CRF85_BC strain in Ningxia may be related to the virus transmission in Henan, China. Additionally, the results of the subsequent phylogenetic tree of the fragment indicates that the emergence of the recombinant strain in Ningxia may also be associated with virus transmission in Henan, China. This highlights the role of population mobility as both a risk factor for HIV transmission and a potential factor in the formation of new strains [18,19].

This study is the first to identify the HIV-1 CRF85_BC recombinant strain in Ningxia, China, and characterize its recombination pattern using near full-length genome sequencing (NFLG). We confirmed it as a second-generation recombinant of CRF85_BC and CRF01_AE, with recombination breakpoints precisely mapped through Simplot and fragmented phylogenetic analysis. These findings provide new insights into HIV-1 genetic diversity in Northwest China. However, the study’s limitations must be acknowledged. While our study identified novel HIV-1 recombinants, the precise timing of recombination events remains uncertain due to limited longitudinal sampling and the inherent challenges of dating recombination events from cross-sectional data. The functionality of the CRF85_BC strain was not evaluated in this study, but previous research has demonstrated that recombination, defined as the exchange of gene fragments between different subtypes or strains, can significantly impact viral replication capacity, drug resistance, and immune escape properties [20,21,22]. Future studies could further assess the replication kinetics and susceptibility to antiretroviral drugs of the CRF85_BC strain through in vitro experiments. The current sample size may constrain the study’s ability to generalize its findings, necessitating expansion to more extensively validate the prevalence and recombination patterns of the CRF85_BC strain in Ningxia.

## 5. Conclusions

This study has identified a CRF01_AE/CRF85_BC recombinant strain, in which CRF85_BC serves as the backbone and two CRF01_AE gene fragments are inserted into the CRF85_BC genomic backbone at loci 7365~8279 and 8431~9492, respectively. The study suggests that multiple exposures to different subtypes, particularly in sexually transmitted populations, are the main factors contributing to the formation of recombinant strains in Ningxia. The identification of these novel recombinants highlights the need to translate genomic surveillance into actionable public health strategies. We recommend the following: (1) integrating phylogenetic cluster analysis with contact tracing programs to prioritize high-risk transmission networks, and (2) combining molecular data with geographic and behavioral metadata to tailor interventions, such as deploying PrEP in identified hotspots. These data-driven approaches are crucial for disrupting emerging HIV transmission chains in Ningxia and similar settings.

## Data Availability

The original data presented in the study are openly available in [National Center for Biotechnology Information, https://www.ncbi.nlm.nih.gov/ (accessed on 26 April 2025)] (Accession numbers PV568063-PV568065).

## Supplementary Materials

The following supporting information can be downloaded at https://www.mdpi.com/article/10.3390/v17050655/s1, Figure S1: Phylogenetic tree of additional HIV-1 strains.

## Abbreviations

The following abbreviations are used in this manuscript:

AIDS	Acquired Immune Deficiency Syndrome
HIV	Human Immunodeficiency Virus
CRFs	circulating recombinant forms
URFs	unique recombinant forms
NFLG	near full-length genome
MSM	men who have sex with men
