# Supplementary material for: Sequencing of One Unique Recombinant CRF85_BC/CRF01_AE Genome and Two Partial Genomes from Ningxia, China
Source: Viruses. 2025 Apr 30;17(5):655. doi: 10.3390/v17050655 (PMC12115780; doi:10.3390/v17050655)
Supplement: Supplementary file 1 [file viruses-17-00655-s001.zip › viruses-3575298-supplementary.pdf]

Supplementary Material

1 Supplementary Figure

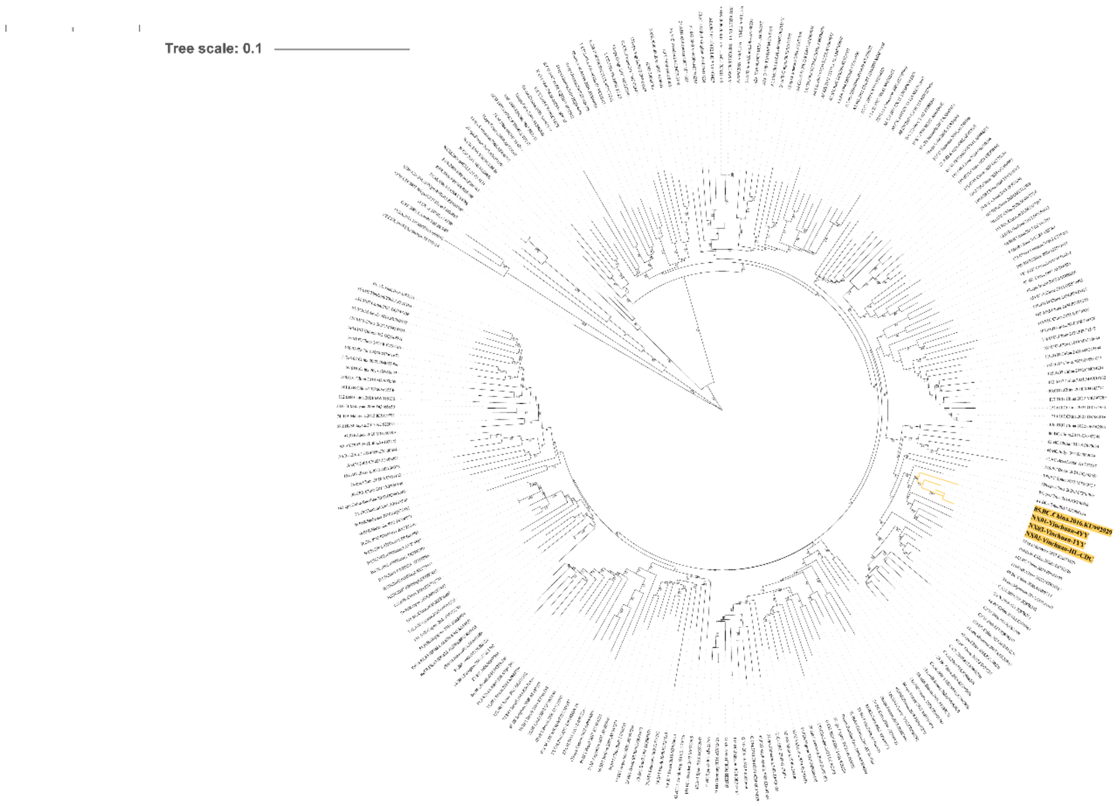

Supplementary Figure S1. Phylogenetic tree of additional HIV-1 strains. The tree includes reference sequences from the Los Alamos HIV Database and highlights the evolutionary position of the CRF85_BC strain identified in this study.
